# Available upon all requests? How and why we should better incentivize the sharing of biomaterials

**DOI:** 10.1371/journal.pbio.3002360

**Published:** 2023-10-06

**Authors:** Suzannah J. Rihn, Alexander Harms

**Affiliations:** 1 Department of Medicine, Cambridge Institute for Therapeutic Immunology and Infectious Disease (CITIID), University of Cambridge, Cambridge, United Kingdom; 2 Department of Health Sciences and Technology (D-HEST), ETH Zurich, Zurich, Switzerland

## Abstract

Could better biomaterial sharing improve the scientific community? This Perspective draws on the authors’ experiences of sharing biomaterials, discussing the advantages and challenges biomaterial sharing creates, and the ways sharing could be more actively promoted and rewarded.

## Teaser

Biomaterial sharing offers enormous benefits for research and for the scientific community. Individuals, funders, institutions, and journals can overcome the barriers to sharing and work together to promote a better sharing culture.

This article is part of the *PLOS Biology* 20th Anniversary Collection.

Most scientists have probably had the experience of reading an inspiring paper, reaching out to the authors about obtaining a new tool or reagent it contains, and then never receiving a response. This can occur despite a biomaterial being listed as “available upon reasonable request”, and at a time when most journals explicitly expect or mandate the sharing of published materials [1, 2]. Yet while there have been concerted efforts to promote the open sharing of data in biology over the past decade, inspired by initiatives and organizations that promote open science, as well as publisher, funder, government, and employer data sharing requirements, the same cannot be said about the sharing of new biomaterials, including the tools or reagents used to generate the open data for publications. Although there have certainly been some notable efforts to encourage biomaterial sharing within specific areas, including large repositories such as Addgene (predominantly for plasmids) [3], as well as smaller, individual, group-led initiatives [4], openly available biomaterial sharing by individual researchers, even following publication, has not yet become the norm.

The reasons reagent requests are commonly ignored, and biomaterials are not openly shared, are not exactly secret, and they do not have to be sinister, although the maintenance of a competitive advantage can certainly have a role. When it comes to the sharing of biological data, such as genome sequences, protein structures, or assay results, their onward sharing may not require much further effort once they are produced and organized for publication and made accessible and discoverable (although hosting can provide issues). However, the onward sharing of biomaterials can prove much more onerous, with many tools or reagents requiring continual researcher effort and funding to produce, manage and distribute. Increasing demands on researchers’ time and resources mean it can be challenging to dedicate time to producing and sending resources to others.

We understand these challenges well, as we have recent, and very positive experiences, of biomaterial sharing. Dr Rihn, and colleagues and collaborators at the MRC-University of Glasgow Centre for Virus Research, University of Dundee, University of Tartu and Griffith University, developed a SARS-CoV-2 and coronavirus toolkit [5], comprising antibodies for nearly every SARS-CoV-2 protein, a single plasmid reverse genetics system, permissive cell lines, and virus isolates. These tools are all openly available, for free or at low cost, and have been globally distributed to hundreds of academic research laboratories, repositories, public health agencies and pharmaceutical companies. Professor Harms generated a representative set of bacteriophages that infect the laboratory workhorse *Escherichia coli* and that can be used as a research tool to efficiently explore the biological diversity of these viruses in any imaginable context. Similar collections are well-established, including for *E*. *coli* itself [6], while a smaller set of seven “T phages” had dominated fundamental molecular biology for decades [7]. The Harms group thus formed the “BASEL collection” (Bacteriophage Selection for your Laboratory), a set of 69 phages that are freely shared with researchers around the world [8], for projects ranging from microbial ecology to bacteriophage therapy.

**Fig 1:**
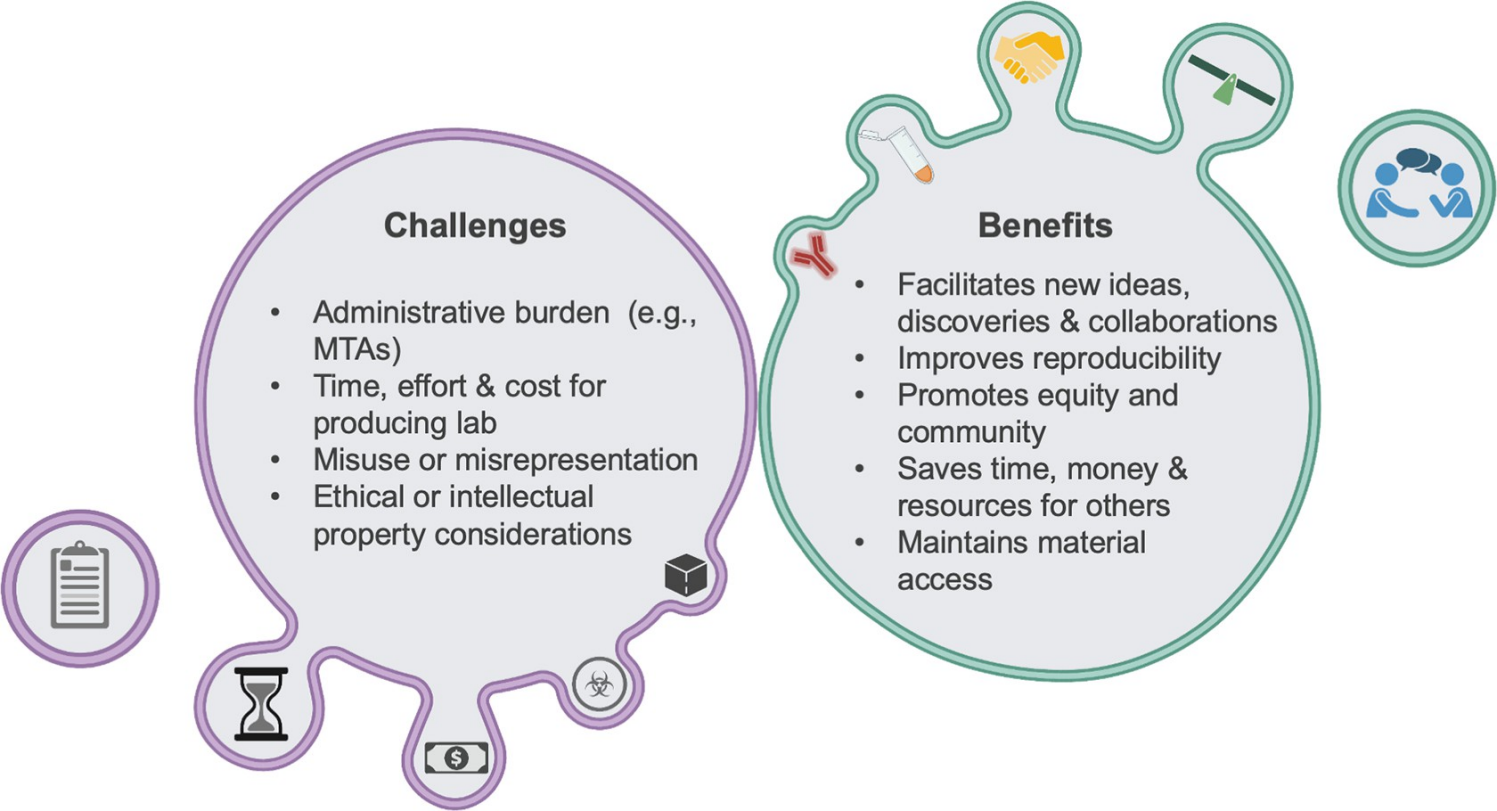
A summary of the benefits and challenges associated with biomaterial sharing. Lists of the key challenges that can hinder biomaterial sharing, as well as the major advantages sharing has to the scientific community. Image created with BioRender.com.

Based on our experiences, we feel able to comment on many of the benefits and challenges concerning biomaterial sharing that we have experienced, which are summarized in Fig 1. It is no secret that academia is reckoning with some deep-rooted systemic issues, ranging from toxic research cultures [9] to unstable funding to reproducibility challenges, amongst others. We believe that improved biomaterial sharing might help ameliorate many of these issues, as our experience and others’ have shown that biomaterial sharing has many benefits.

Perhaps most importantly, by sharing tools and reagents with others, we can facilitate research ideas that we either might not have identified on our own, or not had the time to pursue. Similarly, sharing can open opportunities for more interdisciplinary collaborations, and it supports a diverse range of approaches that could enable unforeseen discoveries or breakthroughs. Crucially, biomaterial sharing also promotes reproducibility by enabling studies to be more directly replicated or compared. Furthermore, when biomaterials are accompanied by thorough protocols and evidence of validation, better standardization can be achieved. Sharing can also lead to valuable savings in time, money and resources. When tools and reagents are shared, other research groups do not need to invest their own efforts or lab funding to replicate biomaterials, allowing researchers to focus on their own investigations. Sharing therefore also offers far better value for funders.

On a more individual level, open sharing of biomaterials can enable scientists to participate in research fields that might otherwise be inaccessible for them. Open biomaterial sharing thus not only encourages positive feelings towards individual colleagues, but it also helps promote a sense of community by showing that science can be an endeavor in which we help each other and work together towards common goals, rather than a competition. Sharing can also help to maintain access to biomaterials when the scientists who developed them move on. Individual laboratory members who generate biomaterials are unlikely to stay in the same lab forever, but by sharing biomaterials, the scientific community can maintain access to, and expertise with, tools that might otherwise be lost. For all of these reasons, more open biomaterial sharing feels essential to the future of academic science.

Yet despite these advantages, there are still substantial barriers to biomaterial sharing. Beyond the obvious challenges of the time and resources required for biomaterial sharing, other factors can impede sharing. For example, material transfer agreements may be required to protect intellectual property and ensure biosafety precautions are employed, but these can sometimes prove time-consuming and restrictive to new research ideas. Sharing can also entail additional time and effort beyond that required to generate and distribute the biomaterials, in order to do things like understand and comply with necessary import and export regulations or troubleshoot protocols for others. Moreover, ethical considerations or restrictions to sharing may apply, if, for example, the materials involve personal data. Equally important are considerations regarding intellectual property, including loss of priority to file possible patents and the according loss of potential income or commercial opportunities. Finally, misrepresentation or other misuse of shared biomaterials could harm their creator, and thereby the scientific community, by degrading trust.

Still, many of these barriers can be avoided or ameliorated by depositing the biomaterials with suitable repositories that can take responsibility for long-term production and distribution challenges. For example, the SARS-CoV-2 permissive cell lines we developed were deposited with the National Institute for Biological Standards and Control (NIBSC) in the UK, and the SARS-CoV-2 viruses we isolated from patient samples were deposited with BEI Resources. Similarly, all phages from the BASEL collection have been deposited at the German Collection of Microorganisms and Cell Cultures (DSMZ). However, the use of repositories is not a perfect solution. Some repositories may still charge prohibitive fees or lack the capacity or skills to manage or distribute certain biomaterials, or be unable to distribute globally. This means equitable access to biomaterials can remain a challenge even when repositories are utilized.

Nevertheless, we, and others [10], think the benefits of biomaterial sharing to both scientific advancement, and to the sense of community amongst scientists, vastly outweigh the challenges. While, in an ideal world, individuals would read the above and feel altruistically inspired to share their biomaterials more openly, we recognize that many of the barriers to biomaterial sharing could preclude this. Still, we believe it is possible for various entities connected to academia to more aggressively promote biomaterial sharing. We therefore suggest that:

- **Funding bodies** create specific schemes to support and encourage biomaterial sharing, to ensure access to tools and skills is maintained.
- **Scientific journals** adopt policies that both more strongly promote biomaterial sharing and foster transparency about authors’ sharing practices following publication.
- **Academic institutions** improve recognition and reward of biomaterial sharing (for example, by including material sharing as part of promotion criteria).
- **Individual researchers** publicize the benefits that biomaterial sharing has had on their research (for example, in talks or on social media).
- **The whole scientific community** of funders, journals, governments, and other institutions, communicate about the benefits that biomaterial sharing create for everyone, not just individuals.
- **All of us** work to normalize open sharing of biomaterials and encourage depositing them in suitable repositories.

Ultimately, we believe that the sharing of biomaterials will be a crucial component in the building of a better scientific community for everyone.
